# Dynamic analysis of skin transcriptome in Xinjiang goats and functional verification of *ELOVL3* in dermal papilla cell proliferation

**DOI:** 10.1016/j.vas.2026.100834

**Published:** 2026-08-24

**Authors:** Cuiling Wu, Qingfa Yan, Sen Tang, Gvlnigar Amar, Asma Anwar, Wenna Liu, Yanqian Wang, Xuefeng Fu

**Affiliations:** a College of Life Science, Xinjiang Normal University, Urumqi, 830017, China; b Xinjiang Uygur Autonomous Region Academy of Animal Science, Urumqi, 830011, China

**Keywords:** Xinjiang goat, Hair follicle growth cycle, RNA-seq, Dermal papilla cells, *ELOVL3*

## Abstract

Xinjiang goats are important dual-purpose livestock, but their cashmere production is affected by environmental variation. The cycle of secondary hair follicles determines cashmere traits, and deciphering its regulatory mechanism under extreme climates is crucial for genetic improvement. Skin samples were collected from Xinjiang goats at six monthly time points across a full annual cycle. RNA-sequencing (RNA-seq) was used to profile gene expression, combined with differentially expressed gene (DEG) analysis and weighted gene co-expression network analysis (WGCNA) to identify key genes and modules associated with follicle cycle and climate factors. Functional validation of *ELOVL3* was performed via immunofluorescence, overexpression, and interference assays in dermal papilla cells (DPCs). The results indicated that RNA-seq yielded high-quality data with 30,579 expressed genes. DEG analysis revealed 853 differential genes in May vs. July, with multiple keratin family genes co-expressed during March–July. WGCNA identified five modules correlated with follicle cycle, temperature, and precipitation. *ELOVL3*, a core gene in the temperature-related module, was expressed in DPCs and promoted DPCs proliferation by accelerating G1/S cell cycle transition. This study characterizes monthly dynamic transcriptional profiles in Xinjiang goat skin, identifies core genes correlated with hair follicle cycle progression and seasonal climatic variation, and verifies the function of *ELOVL3* in promoting dermal papilla cell proliferation. The causal functions of the candidate genes in terms of climate adaptation still require further verification. The results provide theoretical support and candidate genes for cashmere quality improvement and stress-resistant breeding.

## Highlights

• The monthly transcriptome analysis of Xinjiang goat skin revealed that from March to July, it is a critical transitional period for hair follicles to shift from the telogen stage to the anagen stage. During this period, there are coordinated differences in the expression of KRT and KRTAP family genes.
• The WGCNA analysis found that there are a total of five core gene modules closely related to the hair follicle cycle, monthly average temperature, and precipitation. Among them, the MEgreen module has a significant correlation with seasonal temperature changes.
• The *ELOVL3* was identified as a core lipid metabolism gene in the temperature-responsive MEgreen module. Functional experiments demonstrated that *ELOVL3* can accelerate the G1/S cell cycle transition and promote the proliferation of dermal papilla cells.

## 1 Introduction

Xinjiang goats are important dual-purpose livestock breeds in China, exhibiting excellent cashmere and meat production performance. Endowed with strong cold resistance, drought tolerance, and roughage adaptability, they are widely distributed across various regions of Xinjiang. However, due to differences in climate and grassland conditions between northern and southern Xinjiang, there are significant variations in cashmere yield and quality among populations of this breed. Therefore, to effectively protect and efficiently utilize their superior genetic resources, systematic selection should be strengthened in practical domestication to fully tap their genetic potential, thereby continuously improving cashmere production performance and quality.

Cashmere is produced by secondary hair follicles (SHFs). The dynamic transition of the SHFs growth cycle (anagen, catagen, and telogen) directly determines cashmere yield, quality, and growth rhythm. This process is synergistically regulated by gene regulatory networks, endocrine signals, and environmental factors (temperature, light, oxidative stress) (Du et al., 2024; Lin et al., 2022; Song et al., 2025). The climate in Xinjiang varies greatly throughout the four seasons, with significant temperature differences between day and night. The sampling site is located in Qitai County, Xinjiang Uygur Autonomous Region (approximately at 44.07 °N latitude and 89.75 °E longitude). This area has a typical temperate continental arid climate. According to the historical climate data from 2020 to 2022 in the WorldClim database, the average temperature in January is approximately −14.5 °C, and the average temperature in July is approximately 25.2 °C. The annual average precipitation is about 107.6 mm, with most of the precipitation concentrated in summer. Additionally, the daily temperature range in this region is large, and the annual temperature difference exceeds 40 °C (Fick & Hijmans, 2017; Harris et al., 2020). This unique ecological environment may regulate the initiation and transformation of the hair follicle cycle by influencing the microenvironment of hair follicle stem cells, the cell proliferation activity, and the function of the skin lipid barrier. As a result, Xinjiang goats have evolved a hair follicle growth regulation mechanism that is adapted to the local environment. Deciphering this mechanism is of great theoretical and practical significance for the targeted improvement of cashmere quality and the breeding of high-quality breeds.

With the development of high throughput sequencing technology, RNA-seq has become an efficient tool for analyzing dynamic gene expression and identifying functional genes (Hu et al., 2022; Wu et al., 2020; Zhiyun Qin et al., 2025). In recent years, transcriptome expression profiles of SHFs in several cashmere goat breeds have been reported, including Italian cashmere goats (Nocelli et al., 2020), Inner Mongolian cashmere goats (Hu et al., 2022; Su et al., 2020), Pashmina goats (Bhat et al., 2021), Shanbei cashmere goats (Wang et al., 2017), and Jiangnan cashmere goats (Wu et al., 2022). The above research has provided abundant data for the study of follicle growth regulation in cashmere goats. WGCNA can effectively screen core gene modules and key genes associated with target traits, overcoming the limitations of single differential gene analysis. (Wang et al., 2021) used WGCNA analysis to construct an interaction network based on the expression profiles of 12-month-old skin tissues of cashmere goats, and identified 12 hub genes related to cashmere production, including *COL1A1, C1QTNF6, COL1A2*, etc. Gong et al. (2022) conducted a WGCNA analysis on long-haired and short-haired Inner Mongolian cashmere goats and identified nine candidate genes related to different hair types, including *KRT39, KRT74, LOC100861184*, etc.

It is worth noting that numerous studies have shown that genes related to lipid metabolism play a significant role in regulating hair follicle growth (Maier et al., 2011; Seo et al., 2025; Wu et al., 2025). The elongation of very long-chain fatty acids (ELOVL) family is a key regulatory factor in lipid metabolism (Nakamura et al., 2014). Its member *ELOVL3* is mainly involved in the synthesis and metabolism of very long-chain fatty acids and plays important roles in maintaining skin barrier function and hair development (Wang et al., 2023). Early studies in mice found that the *ELOVL3* gene is expressed in five types of hair follicle cells, including dermal papilla cells (DPCs), skin fibroblasts, melanocytes, matrix cells, and outer root sheath cells, with specific high expression in outer root sheath cells (Rendl et al., 2005). However, its function and regulatory mechanism in goat DPCs remain unclear. As the key cells regulating the hair follicle growth cycle, the proliferative activity of DPCs determines the initiation and maintenance of hair follicles (Madaan et al., 2018). Clarifying the regulatory role of *ELOVL3* in DPCs function can provide a new entry point for understanding the mechanism of goat hair follicle growth.

Therefore, the objective of this study is to elucidate the regulatory function of *ELOVL3* in DPCs proliferation and cell cycle progression, and to identify key genes and modules involved in hair follicle cycle regulation and climate adaptation, and provide theoretical basis and candidate gene resources for the genetic improvement of cashmere quality and breed selection of Xinjiang goats.

## 2 Materials and methods

### 2.1 Collection of skin tissue samples

Three healthy female Xinjiang goats of the same age (1 year old) and similar body weight (23.0 ± 1.0 kg) were selected from the Kechuang Breeding Center. All the experimental goats were half-siblings and grew under the same feeding and management conditions, with free access to water and food. The health status of the experimental animals was confirmed to be good before sampling. For these three Xinjiang goats, skin samples were repeatedly collected from the left scapular region every two months, at six time points: in January (X1), March (X3), May (X5), July (X7), September (X9), and November (X11), generating a total of 18 skin tissue samples.

The sampling procedure is as follows: First, completely remove all the hair from the unilateral scapular area, and then disinfect the target skin surface with 75 % medical alcohol and iodophor in sequence. Use a disposable skin biopsy punch device to collect uniform full-thickness skin tissue around the pre-marked scapular area. During each sampling, take another sample 1-2 centimeters away from the previous wound site, while maintaining it within the overall scapular area. This ensures the consistency of the sampling area during the six sampling processes. After tissue collection, disinfect the wound with iodophor again and apply tissue-regenerating powder for wound healing care.

After rinsing with DEPC water, the collected samples were immediately frozen in liquid nitrogen for preservation. Historical weather data (monthly average temperature (MAT), average maximum temperature, average minimum temperature, and total precipitation) of the region were obtained from the WorldClim database, with data acquisition coordinates consistent with that of the geographical location of the Kechuang Breeding Center.

### 2.2 RNA-seq of skin tissues

Total RNA was extracted from 18 Xinjiang goat skin samples using the TRIzol method, following the kit instructions. After extraction, the OD260/280 and OD260/230 ratios of total RNA were measured using a NanoDrop 2000 (Thermo Fisher Scientific Inc., USA) to evaluate RNA purity. The RNA integrity number was determined using an Agilent 2100 (Agilent Technologies Inc., USA). The quality of RNA must meet the requirements for library construction: concentration ≥ 100 ng/µL, total amount ≥ 1 µg, volume ≥ 10 µL, and purity can be judged by the OD_260/230_ ratio being between 1.8 and 2.2, with normal absorption peak at 260 nm, and RNA integrity number (RIN) ≥ 7. After passing quality inspection, the total RNA was entrusted to Biomarker Technologies Corporation for library construction and RNA-seq. Library construction was performed according to the sequencing platform instructions. Paired-end sequencing (PE150) was carried out using the Illumina NovaSeq 6000 platform.

### 2.3 Preprocessing and basic analysis of RNA-seq data

Raw data were quality-assessed using FastQC (v0.11.9) software, and low-quality reads were filtered using Trimmomatic (v0.39) software. After filtering, the clean data from 18 skin tissue RNA-seq samples were aligned to the reference genome of Capra hircus (GCF_001704415.2_ARS1.2_genomic.gff) using Hisat 2.0 software. Transcript assembly and gene expression quantification were performed using StringTie 2.2.0 software to obtain the count values of genome-wide genes. The count values were normalized to counts per million (CPM) values using the cpm function of the edgeR (v4.8.2) R package. Density distribution plots of all samples were drawn using the ggplot package, and principal component analysis (PCA) of samples was performed using the OECloud online tool (https://cloud.oebiotech.com).

### 2.4 Differential expression gene analysis

Differential expression analysis was performed using the standard DESeq2 (v1.50.2) workflow, with the raw count values of known genes from the 18 samples as input. In this analysis, each sample was treated as an independent biological replicate. Differential expression analysis was conducted for six pairs of monthly samples: X1 vs. X3, X3 vs. X5, X5 vs. X7, X7 vs. X9, X9 vs. X11, and X11 vs. X1. Differentially expressed genes (DEGs) were screened with thresholds of adjusted *P* value (padj) < 0.05 and |log_2_ (Fold Change)| > 1. The padj values were calculated using the Benjamini-Hochberg (BH) method implemented in DESeq2 to control the false discovery rate (FDR) for multiple testing. The number of differential genes in each group was counted, and the overlapping distribution characteristics of differential genes among different groups were visualized using a Venn diagram (Bardou et al., 2014).

### 2.5 Weighted gene co-expression network analysis

WGCNA (v1.73) package was used for weighted gene co-expression network analysis with the CPM values of gene expression in 18 samples as input data. Before analysis, low-expression genes were filtered out, and known genes with CPM > 1 in 80 % of the samples were retained to ensure data quality meets the analysis requirements. A clustering tree was drawn based on the correlation between samples, and a pairwise Pearson correlation matrix between all genes was constructed. The pickSoftThreshold function was used to calculate the scale-free topology fit index under different soft thresholds (β). Based on the optimal β value, the pairwise Pearson correlation coefficients between genes were converted into an adjacency matrix to construct a weighted co-expression network. The expression similarity between genes was calculated using the Topological Overlap Matrix, and different co-expression modules were divided with a minimum module size (minModuleSize) of 30. The Pearson correlation coefficient and corresponding significance *P* value between each module eigengene (ME). Bonferroni multiple testing correction was implemented to adjust all raw *P* values and control the family-wise error rate; modules with corrected *P* < 0.05 and |r| > 0.6 were defined as significantly trait-associated core modules. The cytoHubba MCC algorithm in Cytoscape v3.9.1 was adopted to screen hub genes within each significant module.

### 2.6 Function annotation analysis

Extract the common genes between the core module genes and the differentially expressed genes as the candidate functional genes. GO (Gene Ontology) functional enrichment analysis and KEGG (Kyoto Encyclopedia of Genes and Genomes) pathway enrichment analysis of candidate genes were performed using the clusterProfiler (v4.18.4) R package. GO terms and pathways with FDR-adjusted *P* values (Benjamini–Hochberg method) < 0.05 were considered significantly enriched. Functional annotation and Protein–Protein Interaction (PPI) network analysis of candidate genes were performed with reference to the STRING database (https://cn.string-db.org/, v12.0). Hub genes of significant modules and PPI networks were visualized using Cytoscape (v3.9.1) software.

### 2.7 Immunofluorescence detection

Fourth-generation DPCs were selected as the research object. Primary DPCs were isolated from the shoulder skin tissue of Jiangnan cashmere goats by mechanical separation. The isolation and culture methods of the cells refer to previously published studies (Wu et al., 2022). DPCs were seeded in 24-well plates precoated with cell climbing slides. When the cell confluence reaches 60 %, discard the original culture medium and add 1000 μL PBS to gently wash the cells, repeating this process 3 times. Add 1000 μL 4 % paraformaldehyde at room temperature for fixation for 10 min. Discard the fixation solution, wash with PBS for 3 times, each time 5 min. Add 1000 μL 0.1 % Triton X-100-PBS at room temperature for permeabilization for 10 min, wash with PBS for 3 times. Add 1000 μL 5 % normal goat serum blocking solution, incubate at room temperature for 30 min. Discard the blocking solution, add 1:100 diluted ELOVL3 polyclonal antibody (bs-14570R, Bioss), incubate at 37 °C in the dark for 2 h. Discard the antibody dilution solution, wash with PBS in the dark 3 times. Add 1:100 diluted biotin-labeled goat anti-rabbit IgG (SA1074, BOSTER), incubate at 37 °C in the dark for 30 min. Following three further PBS washes, cells were treated with SABC-Cy3 complex diluted at 1:200 and incubated at 37 °C for 30 min under dark conditions. After thorough washing with PBS three times, add the anti-fluorescence quenching agent covering solution containing DAPI staining solution (P0131, Beyotime Biotechnology) to cover the cells; after placing it in the dark for 10 min, collect the images using an inverted fluorescence microscope.

### 2.8 Verification of transfection efficiency

Overexpression vector primers and three groups of small interfering RNA (siRNA) (Supplementary Table S1) for the goat *ELOVL3* gene (XM_005698356) were designed based on the sequence from the NCBI database (https://www.ncbi.nlm.nih.gov/) and synthesized by GenePharma. The restriction enzyme system for overexpression vector construction included BamHI endonuclease and EcoRI endonuclease. All the transfection-related experimental groups were as follows: Blank group (untreated normal DPCs), si-NC group (control of siRNA group), ELOVL3-NC group (empty vector pCDNA3.1(+)), and the targeted treatment groups (si-ELOVL3 fragment or pCDNA3.1(+)-ELOVL3). Lipofectamine™ 3000 (L3000015, Thermo Fisher Scientific) was used to deliver plasmids and siRNAs into fourth-generation DPCs following standard manufacturer protocols.

After 24 hours of transfection, total RNA was collected from each group of cells. Reverse transcription was carried out according to the instructions of the StarScript II First-strand cDNA Synthesis Kit-II (A222-02, GenStar). Realtime fluorescence quantitative PCR (qPCR) was performed in the Bio-Rad CFX96 Real-Time PCR system following the instructions of 2 × RealStar Green Fast Mixture (A301-05, GenStar). The qPCR primer list is provided in Supplementary Table S2. Total proteins from each group of cells were collected 48 hours after transfection, and the overexpression and transfection effects were verified by Western blotting. The dilution ratio of ELOVL3 Polyclonal Antibody and beta-Actin Polyclonal Antibody (bs-0061R, Bioss) was 1:1000.

### 2.9 Detection of cell proliferative activity

For the CCK-8 assay, DPCs were seeded at a density of 5 × 10³ cells per well in a 96-well plate. Each experimental group contained six technical replicate wells. After reacting with CCK-8 reagent (HY-K0301, MedChemExpress) for 2 hours, the OD value was detected at 450 nm using a microplate reader. For EdU proliferation staining, cells were seeded at a density of 1 × 10⁴ cells per well in a 24-well plate, with each group containing three technical replicate slides. The proliferation efficiency was detected using the Edu-488 cell proliferation detection reagent (C0071S, BOSTER) after transfection. For the flow cytometry cell cycle analysis, DPCs were seeded at a density of 1 × 10⁵ cells per well in a 6-well plate, with three technical replicates in each group. Cell proliferation was detected using a cell cycle and apoptosis detection kit (C1052, Beyotime Biotechnology) on a flow cytometer (Beckman Coulter FC500, USA).

All data were expressed as mean ± standard error (SEM). Multiple group comparisons were performed using one-way analysis of variance (One-way ANOVA) combined with Tukey's post hoc multiple comparison test, and two-group comparisons were performed using the independent sample t-test.

## 3 Results

### 3.1 Quality assessment of sequencing data

**Fig. 1:**
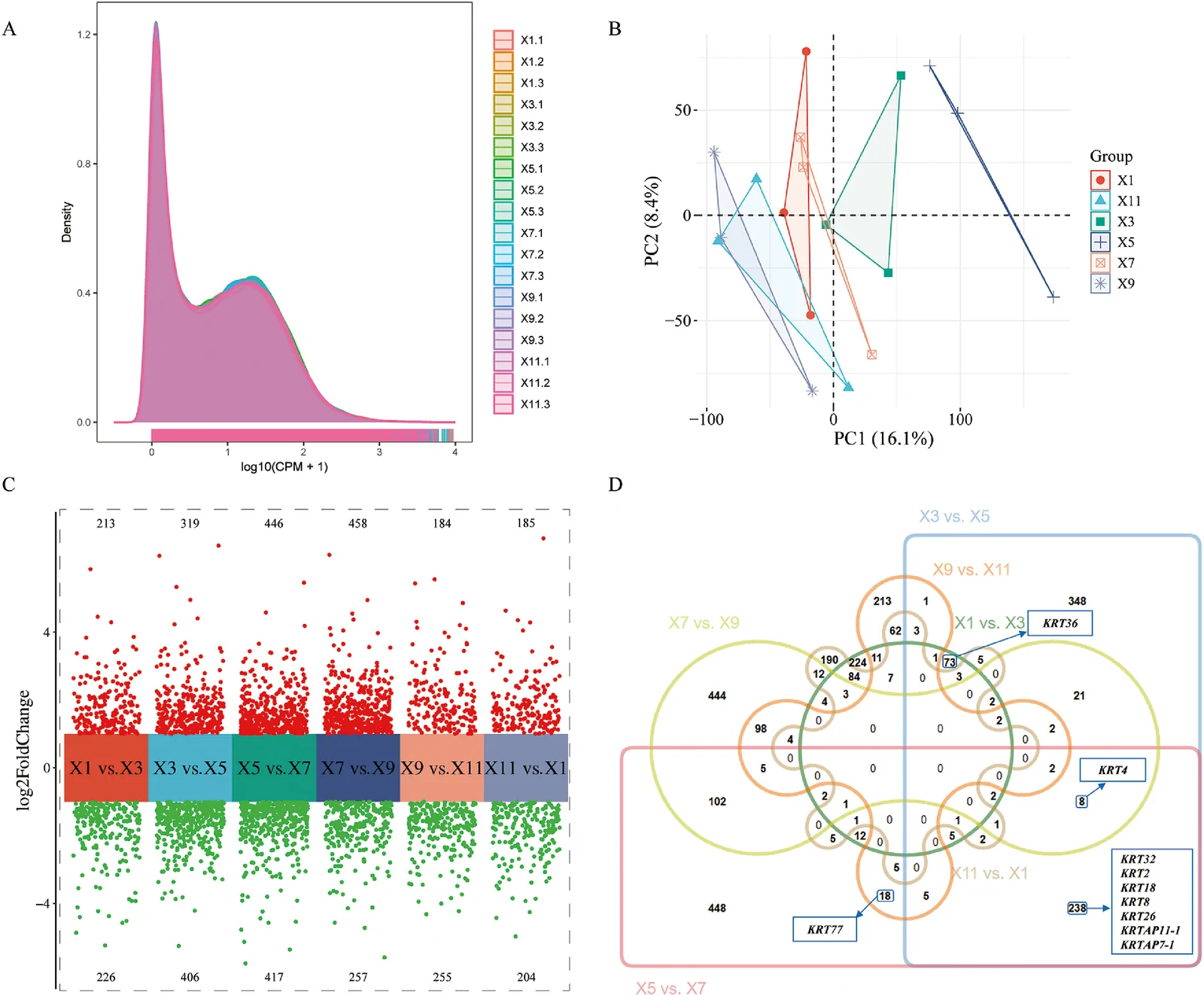
Analysis of sample expression profiles and differential genes. (A) Density distribution plot of gene expression levels. The overlapping curves indicate consistent global expression distributions among samples. (B) Principal component analysis plot of samples. (C) Volcano map of DEGs. Red dots indicate up-regulated genes, green dots indicate down-regulated genes. (D) Venn diagram of DEGs.

After sequencing 18 Xinjiang goat skin tissue samples, the raw data were filtered to obtain clean data. Sequencing data statistics showed that a total of 621.84 M Reads and 185.97 Gb Clean Data were obtained from the 18 samples. The Clean Data of each sample reached 9.54 Gb, and the Q30 base percentage was above 95.02 % (Supplementary Table S3). The mapping rates of all samples to the reference genome ranged from 94.80 % to 96.43 %, indicating that the quality of the sequencing data meets the experimental standards (Supplementary Table S4). After filtering low-expression genes (retaining genes with Count value ≥ 10 in at least 6 samples), a total of 30,579 gene expression profiles were obtained. The density distribution plot showed that the curves of each sample overlapped highly, indicating that the overall distribution pattern of gene expression levels in different samples was consistent, and the experimental reproducibility was reliable (Fig. 1A). The PCA scatter plot showed that the X9 and X11 groups had small expression differences. Samples from other groups showed a certain clustering trend according to the sampling month, with some dispersion of replicate points within the group, but still within a reasonable range, indicating that the experimental data were overall credible (Fig. 1B).

### 3.2 DEG analysis

The DEG analysis results showed that a total of 439 known differential genes were obtained in the X1 vs. X3 group (213 up-regulated and 225 down-regulated). The X3 vs. X5 group had 725 differential genes (319 up-regulated and 406 down-regulated), the X5 vs. X7 group had 853 differential genes (446 up-regulated and 417 down-regulated), the X7 vs. X9 group had 715 known differential genes (458 up-regulated and 257 down-regulated), the X9 vs. X11 group had 439 differential genes (184 up-regulated and 225 down-regulated), and the X11 vs. X1 group had 389 differential genes (185 up-regulated and 205 down-regulated) (Fig. 1C, Table 1). The Venn diagram showed that multiple keratin (KRT) and keratin-associated protein (KRTAP) genes were differentially expressed in multiple groups. *KRT36* was commonly differentially expressed in X1 vs. X3 and X3 vs. X5, and KRT4 was commonly differentially expressed in X3 vs. X5, X5 vs. X7, and X7 vs. X9. *KRT32, KRT2, KRT18, KRT8, KRT26, KRTAP11-1*, and *KRTAP7-1* were commonly differentially expressed in X3 vs. X5 and X5 vs. X7 (Fig. 1D).

**Table 1:** Summary of differentially expressed genes (DEGs) in pairwise comparisons of skin tissues from Xinjiang goats across different months.

Comparison	Total DEGs	Up-regulated	Down-regulated
X1 vs. X3	439	213	225
X3 vs. X5	725	319	406
X5 vs. X7	853	446	417
X7 vs. X9	715	458	257
X9 vs. X11	439	184	225
X11 vs. X1	389	185	205

### 3.3 Climatic data and network construction parameters

**Fig. 2:**
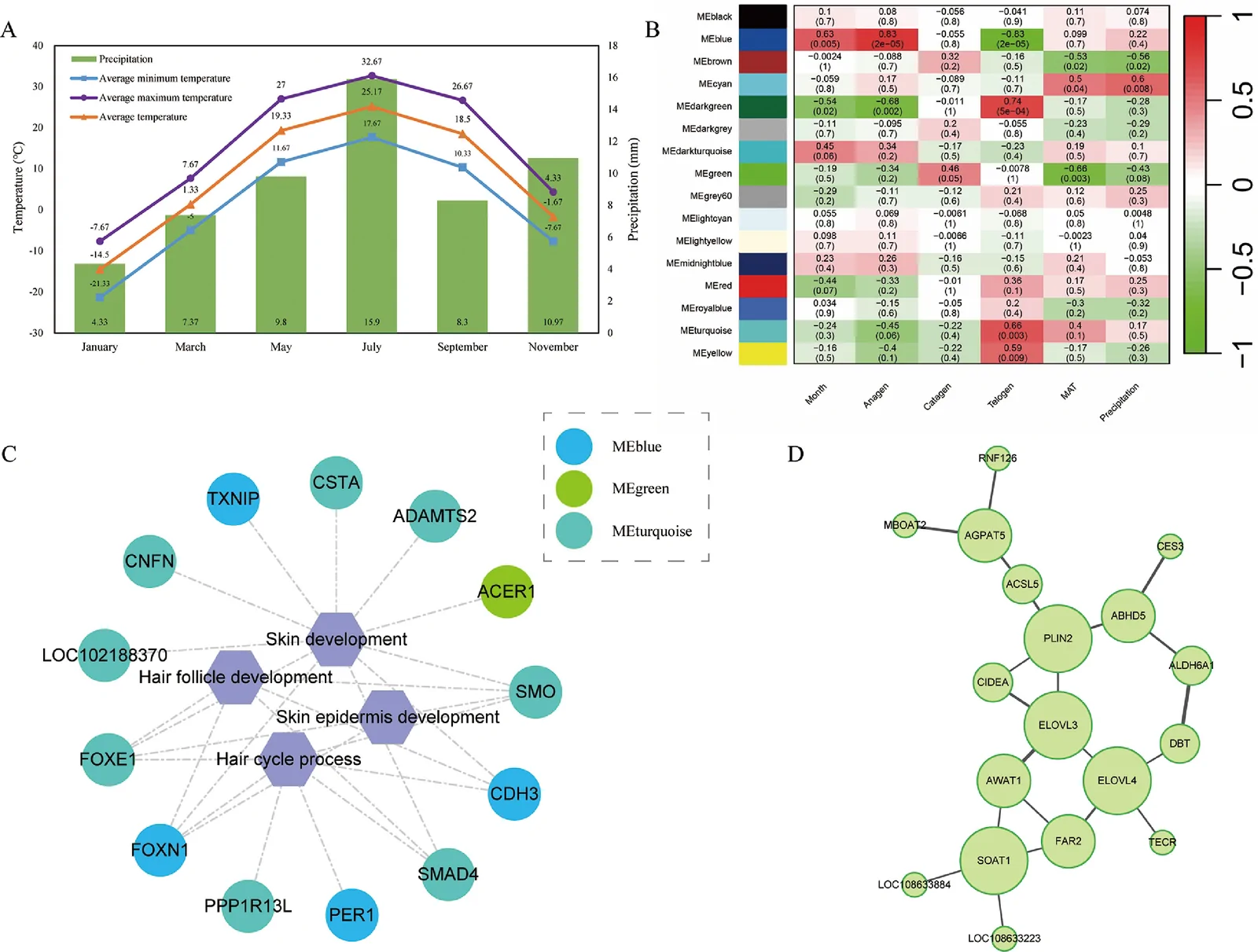
WGCNA analysis and functional annotation. (A) Statistical chart of climatic data for six sampling months in the sampling area; (B) Module-trait correlation heatmap. The number in each grid represents the correlation between the module and the trait, and the number in parentheses represents the significance *P* value. MAT represents monthly average temperature. (C) Candidate genes and functional network. The circles represent genes and hexagons represent functions. (D) MEgreen protein-protein interaction network. Node size reflects the degree of connectivity; larger nodes indicate higher connectivity.

Statistical analysis of climatic data for the 6 sampling months in the sampling area (Qitai County, Xinjiang) showed that the average temperature (−14.5 °C) and precipitation (4.33 mm) in January were the lowest, while the average temperature (25.17°C) and total precipitation (15.9 mm) in July were the highest (Fig. 2A). Climatic factors showed obvious seasonal fluctuation characteristics. Following the WGCNA soft-thresholding selection criteria (Langfelder & Horvath, 2008; Zhang & Horvath, 2005), we selected β = 4 to construct the network. At this power, the scale‑free topology fit index reached R² > 0.9, and the mean connectivity remained reasonably high (Supplementary Figure S1A). After screening, 14,754 known genes were used for the WGCNA analysis. Modules with highly similar expression patterns were merged with a merging threshold of 0.25, resulting in 16 co-expression modules. Each module was represented by a different color. The MEturquoise contained the largest number of genes (3,312), while the MEdarkgrey contained the smallest number (53) (Supplementary Figure S1B).

### 3.4 Module-trait correlation analysis and core gene screening

A module-trait correlation heatmap was constructed using module information and phenotypic traits. With thresholds of *P* < 0.05 and absolute correlation coefficient (|r|)> 0.6, a total of five significantly correlated modules were obtained (Fig. 2B). Among these modules, the MEblue (containing a total of 3,012 genes) showed extremely significantly correlated with Month (r = 0.63), Anagen (r = 0.83), and Telogen (r = −0.83) (*P* < 0.01). The MEcyan (115 genes) was extremely significantly correlated with monthly total precipitation (r = 0.60) (*P* < 0.01). The MEdarkgreen (72 genes) was extremely significantly correlated with Anagen (r = −0.65) and Telogen (r = 0.74) (*P* < 0.01). The MEgreen (234 genes) was extremely significantly correlated with MAT (r = −0.66, *P* < 0.01). The MEturquoise (3,312 genes) was extremely significantly correlated with Telogen (r = 0.66, *P* < 0.01). Hub genes were screened using the Maximal Clique Centrality (MCC) algorithm of the cytoHubba plugin in Cytoscape (v3.9.1) software. The top 10 hub genes of each significant module are shown in Supplementary Figure S2. The temporal expression dynamics of the top 10 core genes in each significant co-expression module are visualized through Supplementary Figure S3. The expression fluctuation patterns of the MEblue, MEcyan, MEdarkgreen, MEdarkgreen, MEgreen and METurquoise modules vary in different months.

### 3.5 Functional annotation analysis

The intersection of differentially expressed genes and significant module genes was conducted, and 1,536 candidate genes were identified. The GO functional annotation and KEGG pathway enrichment analysis were performed on these candidate genes. The candidate genes were significantly enriched in 48 GO terms, mainly including Intermediate filament (GO: 0005882), Intermediate filament cytoskeleton (GO: 0045111), and Supramolecular complex (GO: 0099080). The top five GO terms with the highest enrichment levels in biological process, cellular component, and molecular function categories were plotted as bubble charts (Supplementary Figure S4A). Only five GO terms were significantly enriched in the molecular function category. Further analysis revealed that four genes (*CDH3, FOXN1, PER1*, and *TXNIP*) in the MEblue, *ACER1* gene in the MEgreen, and eight genes (*FOXE1, PPP1R13L, SMO, SMAD4, ADAMTS2, CNFN, CSTA*, and *LOC102188370*) in the MEturquoise were associated with skin development and hair follicle cycle growth (Fig. 2C).

KEGG pathway enrichment analysis results showed that all candidate genes were significantly enriched in 10 pathways. The top three pathways were Staphylococcus aureus infection (chx05150), Estrogen signaling pathway (chx04915), and Tuberculosis (chx05152) (Supplementary Figure S4B). In the protein-protein interaction network composed of 18 genes in MEgreen, *PLIN2, ELOVL3, ELOVL4*, and *SOAT1* genes were core node genes (Fig. 2D). These genes were mainly annotated in biological processes such as lipid droplet regulation, fatty acid metabolism, and lipid synthesis. It is speculated that the green module may be involved in regulating hair follicle growth and development or skin adaptation to seasonal environmental changes by regulating lipid metabolism pathways.

### 3.6 *ELOVL3* expression verification

**Fig. 3:**
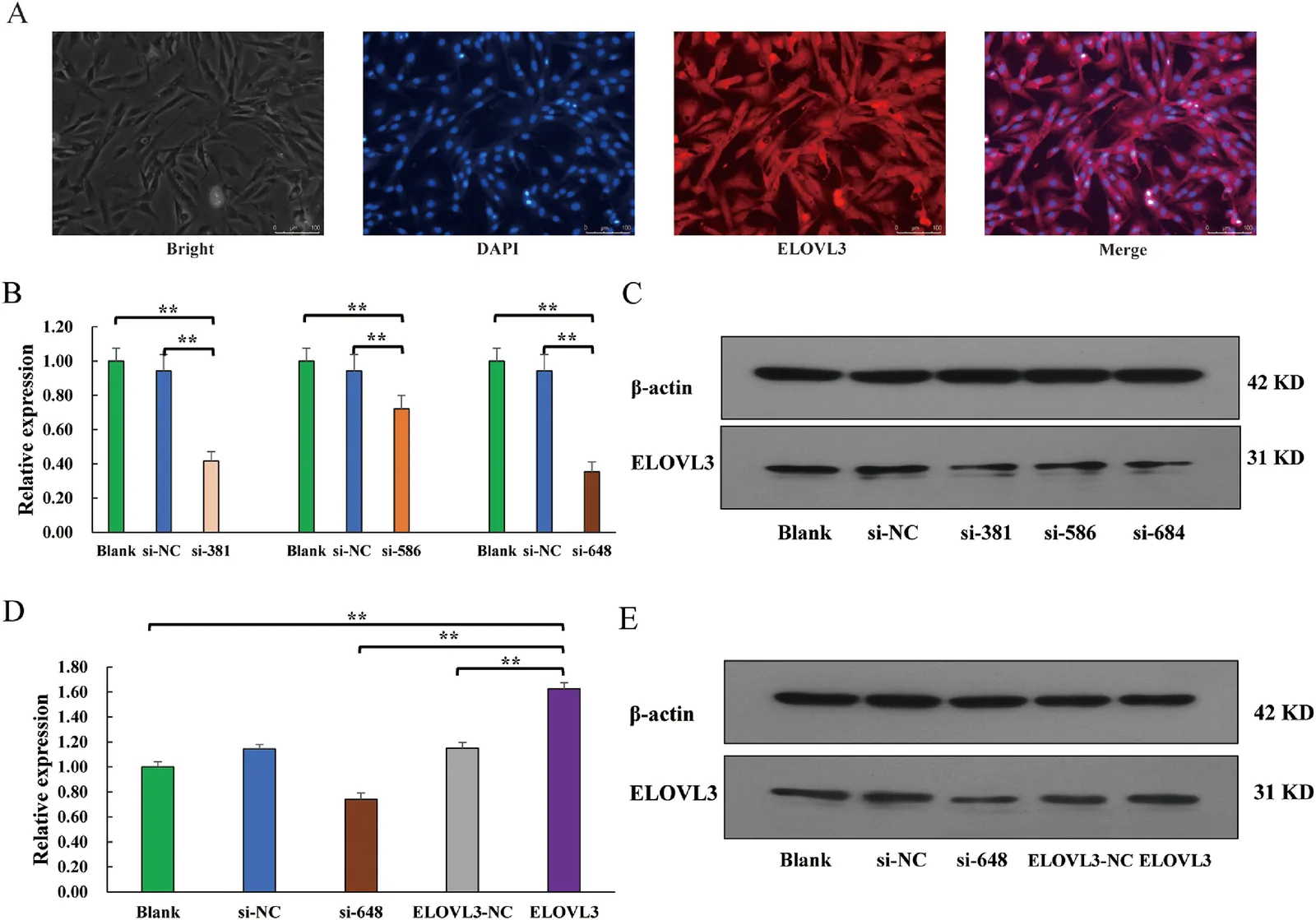
The verification of ELOVL3 expression. (A) Identification of ELOVL3 expression in DPCs. (B) Screening of *ELOVL3* gene interference fragments based on mRNA expression levels. (C) Screening of ELOVL3 interference fragments based on protein expression levels. (D) Detection of *ELOVL3* gene overexpression efficiency at the mRNA level. (E) Detection of ELOVL3 overexpression efficiency at the protein level. Data were expressed as mean ± SEM, and T-test was used for statistical analysis. ** represents extremely significant difference, *P* < 0.01.

Immunofluorescence staining results demonstrated that ELOVL3 was abundantly expressed in the cytoplasm of cashmere goat DPCs, with faint positive signals also detectable within the cell nucleus (Figure A). RT-qPCR and Western Blot results confirmed that si-648 was selected as the *ELOVL3* gene siRNA for subsequent experiments (Fig. 3B, C). The cDNA sequence of the goat *ELOVL3* gene was successfully cloned, with a full length of 825 bp. The eukaryotic expression vector of the *ELOVL3* gene, pCDNA3.1(+)-ELOVL3, was constructed (Supplementary Figure S5). RT-qPCR detection showed that the overexpression efficiency of the *ELOVL3* gene was 162.50 ± 12.30 %, and the inhibition efficiency of the si-648 was 74.10 ± 8.70 % (Fig. 3D). Western Blot detection showed that the expression pattern of ELOVL3 protein was consistent with that of mRNA (Fig. 3E). The above results indicated that both the *ELOVL3* overexpression vector and interference fragment were successfully transfected into DPCs, and the transfection efficiency met the requirements of subsequent functional verification.

### 3.7 Effect of *ELOVL3* gene on DPCs proliferative activity

**Fig. 4:**
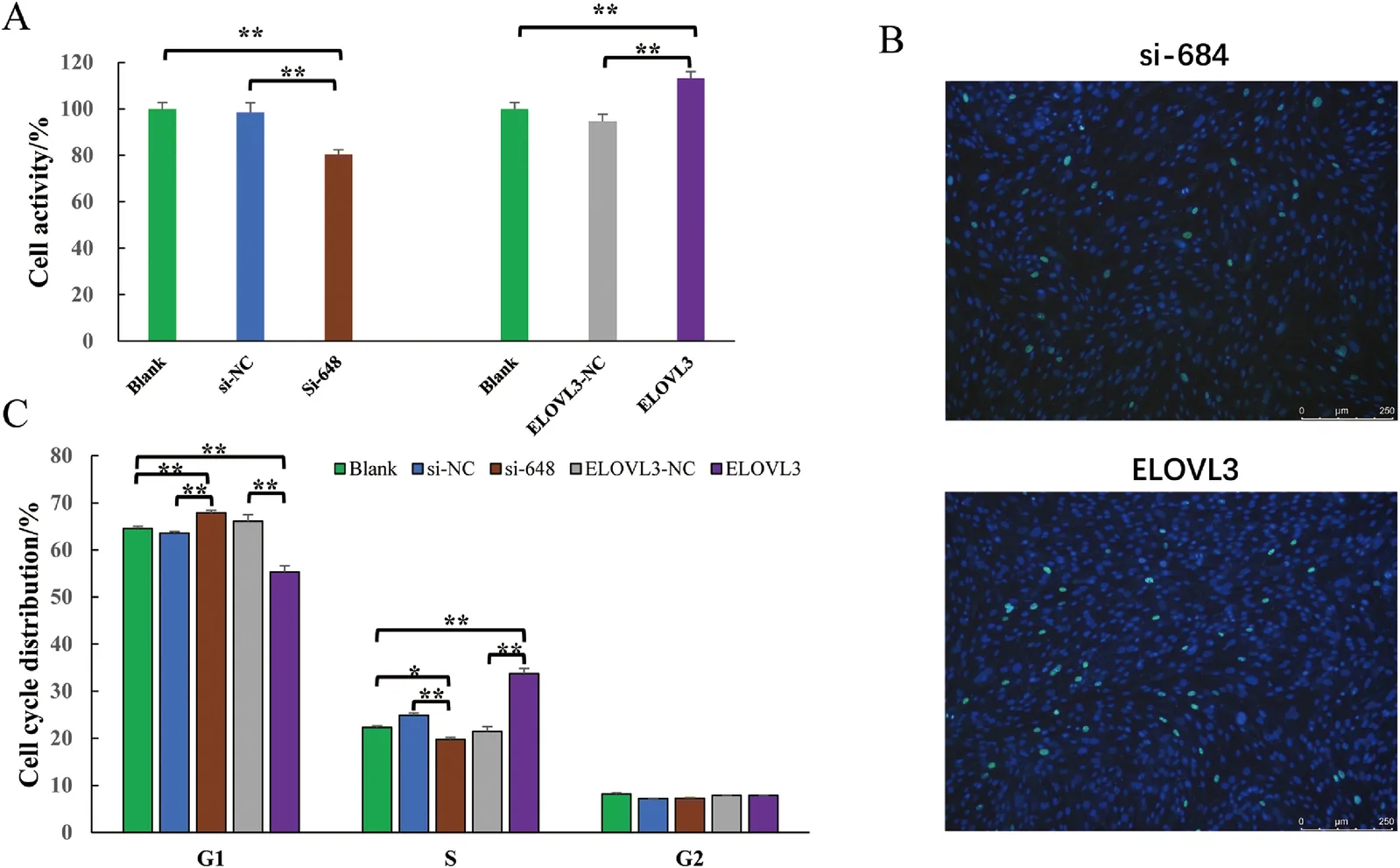
Effect of *ELOVL3* gene on dermal papilla cell proliferation. (A) CCK-8 assay for cell activity. (B) Edu assay for cell proliferation. (C): Flow cytometry assay for cell cycle. Data were expressed as mean ±SEM, One-way ANOVA combined with Tukey’s post-hoc multiple comparison test was used for datasets containing three or more experimental groups. For analyses with only two experimental groups, independent sample t-test was utilized. * denotes a significant difference at *P* < 0.05, and ** denotes an extremely significant difference at *P* < 0.01.

Using the Blank group as a control, CCK-8 assay showed that the cell activity of the si-NC group was 98.45 %, the si-648 group was 80.42 %, the overexpression NC group was 94.67 %, and the overexpression group was 113.08 %. Compared with the Blank group and the si-NC group, the cell activity of the si-648 group was extremely significantly decreased (*P* < 0.01). Compared with the Blank group and the ELOVL3-NC group, the DPCs activity of the ELOVL3 group was extremely significantly increased (*P* < 0.01) (Fig. 4A). EDU assay showed that the number of proliferating DPCs in the ELOVL3 group was higher than that in the si-648 group (Fig. 4B). Flow cytometry analysis showed that compared with the Blank group and the si-NC group, the proportion of cells in the G1 phase in the si-648 group was extremely significantly increased (*P* < 0.01), the proportion of cells in the S phase was significantly decreased (*P* < 0.05), while the proportion of cells in the G2 phase showed no significant change (*P* > 0.05). The results indicate that interfering with the expression of *ELOVL3* inhibits the cells from entering the S phase from the G1 phase. Compared with the Blank group and the ELOVL3-NC group, the proportion of cells in the G1 phase in the ELOVL3 group was extremely significantly decreased (*P* < 0.01), the proportion of cells in the S phase was extremely significantly increased (P < 0.01), and there was no significant change in the proportion of cells in the G2 phase (*P* > 0.05). The results indicate that overexpression of *ELOVL3* promotes the cells to enter the S phase from the G1 phase (Fig. 4C).

## 4 Discussion

Hair follicles are small organs in the skin, and their growth, development, and cyclic turnover depend on the precise regulation of skin microenvironment homeostasis. Seasonal environmental factors such as temperature and precipitation throughout the year affect hair growth. The extreme climatic conditions in Xinjiang have provided unique selective pressures for the formation of the quality of goat hair and the evolution of environmental adaptability. This study conducted RNA-seq sequencing, DEG analysis, WGCNA and candidate gene function verification on the skin tissues of Xinjiang goats at six key time points throughout the year. It systematically explored the dynamic changes in gene expression in the skin tissues of Xinjiang goats, revealed the potential molecular mechanisms regulating the hair follicle growth cycle and climate adaptability, and provided theoretical basis and candidate gene resources for the genetic improvement of cashmere quality in Xinjiang goat.

### 4.1 Differential expression of multiple KRT or KRTAP genes

Hair follicle growth and development is a complex process regulated by multiple genes synergistically. With the cyclic turnover of secondary hair follicles in cashmere goats throughout the year, the gene expression pattern in the skin also changes. DEG analysis of six pairs of monthly samples showed that the number of differential genes was the highest (853) in the skin tissues between May and July (X5 vs. X7 group), which was highly consistent with the growth and development law of secondary hair follicles in cashmere goats. Specifically, March to May is the telogen phase of secondary hair follicles in Xinjiang goats, and July marks the entry into the anagen phase. Meanwhile, seven genes (*KRT32, KRT2, KRT18, KRT8, KRT26, KRTAP11-1*, and *KRTAP7-1*) were differentially expressed in both X3 vs. X5 and X5 vs. X7 groups, further confirming that March–July is a critical transition period for hair follicle growth and development in Xinjiang goats (Fig. 1D).

Keratin family genes are core structural genes composing hair follicles and hair fibers (Ledford et al., 2022). Among them, *KRT26* and *KRT32* are hair/hair follicle-specific keratins, mainly involved in the formation of the hair cuticle and inner root sheath, and their differential expression could directly affect the assembly and structural stability of hair fibers (Moll et al., 2008). *KRT2* has been previously confirmed to be differentially expressed in skin of different colors and plays an important role in coat color regulation (Cui et al., 2016; Fan et al., 2013). *KRT8* and *KRT18* are single-layer epithelial-specific keratins, involved in maintaining the integrity of the cytoskeleton of epithelial cells around hair follicles and providing structural support for hair follicle growth (Ho et al., 2022). *KRTAP11-1* and *KRTAP7-1* belong to high glycine-tyrosine keratin-associated proteins, which enhance the mechanical strength and toughness of hair by cross-linking with keratin intermediate filaments (Fujimoto et al., 2014; Ullah et al., 2020). During the anagen phase of hair follicles, when cells in the hair bulb rapidly differentiate into cortical or cuticular hair keratinocytes, a large number of keratin genes are transcriptionally activated. The synergistic differential expression of these genes during March–July suggests that they may jointly promote the transition of secondary hair follicles from the telogen to the anagen phase and the development of hair fibers by regulating the keratinization process of hair follicles and maintaining the structural stability of hair follicles.

### 4.2 WGCNA identified genes related to the follicular cycle

In this study, WGCNA identified five modules significantly associated with the hair follicle cycle (anagen and telogen), MAT, and precipitation traits (*P* < 0.05, |r| > 0.6), providing a basis for the precise screening of gene modules regulating core traits. Among them, the MEcyan was extremely significantly correlated with precipitation traits. The six hub genes (*LOC108638285, LOC108636561, LOC108636552, LOC108638293, LOC108638290, LOC108635497*) in this module belong to members of the KRTAP family (Supplementary Figure S2B). Their expression levels were significantly increased when precipitation was the highest in July. There is a strong correlation between precipitation and the MEcyan module rich in KRTAP family genes. This leads us to propose an unverified hypothesis: these KRTAP members may be involved in maintaining the moisture of the hair follicle cuticle and the skin barrier function in response to changes in environmental humidity. It should be emphasized that the differences in gene expression between different sampling months do not prove that they have a direct regulatory function in response to precipitation. Therefore, further functional experiments are necessary to verify this hypothesis.The three modules MEblue, MEdarkgreen, and MEturquoise were extremely significantly correlated with hair follicle cycle traits (*P* < 0.01), indicating that there are core genes regulating the transition of the hair follicle growth cycle in Xinjiang goats in these modules. We noticed that four hub genes (*KIF2C, CLSPN, NUF2*, and *NEK2*) in MEblue could be annotated to the Mitotic cell cycle process (GO: 1903047) GO Term (Supplementary Figure S2A). As cell cycle marker genes, these four genes have been confirmed to be used for cell cycle staging in single-cell RNA sequencing data, providing key indicators for explaining the transition between proliferation and quiescent states during the differentiation of hair follicle dermal condensate cells (Gupta et al., 2019; Nestorowa et al., 2016). Combined with DEGs and GO functional annotation, *TXNIP, CDH3, FOXN1*, and *PER1* in MEblue were identified as candidate genes. The *TXNIP* regulates cellular redox homeostasis by interacting with the antioxidant protein thioredoxin (Gong et al., 2025; Hsiao et al., 2022). Our early study found that the expression level of the *TXNIP* gene in ultra-fine wool sheep was higher than that in fine wool sheep, and fiber diameter is one of the important indicators for evaluating wool quality (Tian et al., 2017). The *CDH3* encodes P-cadherin, which is involved in cell-cell adhesion. Pathogenic mutations in this gene can lead to hypotrichosis in humans (Al Zubi et al., 2022; Samuelov et al., 2015). As a transcription factor expressed in the epidermis, *FOXN1* has been confirmed to play a key role in the differentiation of keratinocytes and hair follicle cells, and is currently used as a target for the treatment of male pattern baldness (Mecklenburg et al., 2001; Potter et al., 2011; Zhu et al., 2025). The circadian clock gene *PER1* affects the hair follicle growth cycle by regulating the cell cycle rhythm, and its knockdown can significantly prolong the anagen phase of hair follicles (Al-Nuaimi et al., 2014; Hardman et al., 2015).

In MEturquoise, eight genes (*FOXE1, PPP1R13L, SMO, SMAD4, ADAMTS2, CNFN, CSTA*, and *LOC102188370*) are regarded as candidate genes. As a downstream target of the Shh/Gli signaling pathway, *FOXE1* regulates the directional growth of hair follicles into the dermis, and its deletion can lead to disordered hair follicle arrangement and thin and curly hair (Brancaccio et al., 2004). Children with biallelic inactivating mutations in the *PPP1R13L* gene show skin and hair abnormalities (Tulbah et al., 2024). The *SMO* is a key component of the hedgehog signaling pathway. The loss of its function leads to the obstruction of dermal aggregates formation and the stasis of hair follicle development. Furthermore, mutations in the *SMO* gene have been reported to cause basaloid follicular hamartomas (Atzmony et al., 2022; Woo et al., 2012). The *SMAD4* is a core regulatory factor of the TGF-β signaling pathway. It has been confirmed in humans, rats and mice that the loss of *SMAD4* function hinders the differentiation and periodic activity of hair follicles, thereby leading to progressive hair loss (Bazzi et al., 2005; Owens et al., 2008; Qiao et al., 2006). The *ADAMTS2* is mainly expressed in fibroblasts and mesenchymal cells, and its loss of activity causes extreme skin fragility (Leduc et al., 2021). The *CNFN* has been identified as a protein component of epidermal keratinocytes, which ensures the structural homeostasis of hair follicles by maintaining the adhesive function of tight junctions in the stratum corneum (Leclerc et al., 2009; Wagner et al., 2019). As a secretory factor enriched in keratinocytes, *CSTA* is not only involved in the regulation of skin aging, but its mutations can also cause Acral peeling skin syndrome (Has, 2018; Liang et al., 2022; Sarika et al., 2021). The *LOC102188370* (*GPR89A*) mainly functions to encode a G protein-coupled receptor, and this gene is annotated in the GO term of Skin development (GO: 0043588). Its role in hair follicle morphogenesis and development has rarely been reported.

It is worth noting that in the monthly sampling, season, hair follicle cycle stage, and meteorological factors (temperature and precipitation) are all highly linearly correlated confounding variables (Hu et al., 2022). Under the unique climate conditions of Xinjiang, temperature and precipitation fluctuate significantly between different months. This occurs simultaneously with the seasonal changes in the hair follicle transitioning from the quiescent phase to the active phase. The module - phenotype correlation derived from the WGCNA analysis only represents a statistical association, not an exact causal relationship. Based on the current observed transcriptome data, we cannot completely separate the independent contributions of hair follicle stages and climate variables to the genetic expression variations. Therefore, the adaptive functions of these hub genes still need to be verified through further experiments in the future.

### 4.3 Regulatory function of the *ELOVL3* gene

The MEgreen module was extremely significantly correlated with MAT traits (*P* < 0.01). Combined with the extreme climatic characteristics of Xinjiang, such as large diurnal temperature differences, cold winters, and hot summers, it is speculated that the genes in this module may be involved in the potential transcriptional regulation of skin tissue adaptation to temperature stress. Protein-protein interaction network analysis showed that the differentially expressed genes *PLIN2* (Roberts et al., 2023; Wu et al., 2025), *ELOVL3* (Qin et al., 2023), *ELOVL4* (Nakamura et al., 2014), and *SOAT1* (Fu et al., 2024) in the green module are core node genes (Fig. 2D), and these genes are mainly involved in biological processes such as lipid droplet regulation, fatty acid metabolism, and lipid synthesis.

In the process of evolution, organisms gradually adapt to various environmental temperature changes to optimize energy allocation. At low temperatures, organisms need more energy to maintain basic physiological functions (Wu et al., 2023). Exposure to low-temperature environments usually leads to increased energy consumption, especially through enhanced fatty acid oxidation to generate additional heat (Rosina et al., 2022). As the first line of defense against external environmental stress, the integrity of the skin lipid barrier directly affects the ability of animals to adapt to extreme temperatures (Hui-Beckman et al., 2023; Shi et al., 2015).

Abnormal lipid metabolism can lead to hair follicle growth disorders. The expression of *ELOVL3* in skin tissues is closely related to skin barrier function and hair development (Guillou et al., 2010). (Westerberg et al., 2004) discovered that mice lacking the *ELOVL3* gene exhibited phenotypes such as sparse hair, inability to resist water, hyperplasia of the sebaceous gland system in hair follicles, and increased loss of epidermal moisture. Early studies found that the *ELOVL3* gene showed a strong diurnal expression pattern only in the liver of sexually mature male mice (Brolinson et al., 2008). Zhou et al. (2018) discovered through transcriptome sequencing that the *ELOVL3* gene was differentially expressed in the skin tissues of Shanbei white cashmere goats at SHFs different stages, which is similar to the results of this study.

This study confirmed through overexpression and interference experiments that *ELOVL3* can significantly affect the proliferative activity of DPCs. Combined with the correlation between the MEgreen and temperature traits, it is speculated that the *ELOVL3* gene may regulate cashmere growth and environmental adaptability in Xinjiang goats through a dual mechanism. On the one hand, it affects the transition of the hair follicle growth cycle by regulating the proliferative activity of DPCs, providing core support for hair development. On the other hand, it maintains the integrity of the skin lipid barrier by participating in lipid synthesis and metabolism, enhancing the adaptability of goats to extreme temperatures in Xinjiang.

Although this study provides valuable transcriptomic profiles of dynamic hair follicle cycles, due to the lengthy experimental period and high costs associated with large‑animal experiments, only three animals were used as biological replicates at each time point. We recognize that this limited sample size inevitably reduces statistical power and may restrict the generalizability of our conclusions. Furthermore, each sample was treated as an independent unit in our DESeq2 analysis, and the repeated‑measures structure arising from serial sampling of the same animal across different time points was not explicitly modeled. In the future, increasing the sample size and conducting in vivo functional experiments will be crucial for ultimately verifying the roles of candidate genes such as *ELOVL3* in hair follicle cycle regulation and climate adaptation.

## 5 Conclusions

This study confirmed through RNA-seq and bioinformatics analysis that the gene expression in the skin of Xinjiang goats has significant monthly dynamic characteristics. March–July is a critical period for secondary hair follicles to enter the anagen phase from the telogen phase. Keratin family and keratin-associated protein genes are synergistically differentially expressed during this stage, regulating hair follicle keratinization and hair fiber development. WGCNA identified five core co-expression modules that showed significant correlations with hair follicle cycle, temperature, and precipitation. Eight potential candidate genes, including *TXNIP, CDH3, FOXN1, PER1, FOXE1, PPP1R13L, SMO*, and *SMAD4*, were screened and speculated to be associated with seasonal hair follicle cycle transitions in Xinjiang goats. As a key gene in the lipid metabolism pathway, *ELOVL3* is the hub gene of the MEgreen module and can promote the proliferation of DPCs by regulating the cell cycle process. These findings indicate that *ELOVL3* is a potentially important candidate gene for regulating the hair follicle cycle cycle in Xinjiang goats. The results of this study provide important theoretical support and candidate targets for the genetic improvement of cashmere quality and breeding of stress-resistant goats in Xinjiang.

## Ethical approval

Sample collection was carried out under license in accordance with the Guidelines for Care and Use of Laboratory Animals of China and all study was approved by the Animal Care and Use Committee of Xinjiang Academy of Animal Science (Approval number 2020018). The experimental animals were cared for after the sampling was completed.

## Ethics declaration

This study was conducted in accordance with the ARRIVE (Animal Research: Reporting of In Vivo Experiments) guidelines. This study was approved by the Institute of Animal Science, Xinjiang Academy of Animal Sciences. (Approval No. 2020018).

## Declaration of generative AI and AI-assisted technologies in the writing process

We declare that this manuscript is entirely our own original work. No generative AI tools or AI-assisted technologies were used in the writing or preparation of this manuscript.

## Funding

This research was funded by the Xinjiang Uygur Autonomous Region Natural Science Foundation Project (Grant No. 2025D01E24), the National Natural Science Foundation of China (Grant No. 32360814), and the Xinjiang Uygur Autonomous Region “Tianshan Talents” training program (Grant No. 2023TSYCCX0031).

## CRediT authorship contribution statement

**Cuiling Wu:** Writing – original draft, Visualization, Supervision, Software, Methodology, Data curation, Conceptualization. **Qingfa Yan:** Writing – review & editing, Data curation. **Sen Tang:** Investigation. **Gvlnigar Amar:** Methodology, Data curation. **Asma Anwar:** Software. **Wenna Liu:** Validation. **Yanqian Wang:** Validation. **Xuefeng Fu:** Writing – review & editing, Supervision, Resources, Project administration, Funding acquisition, Formal analysis.

## Declaration of competing interest

The authors declare the following financial interests/personal relationships which may be considered as potential competing interests: Xuefeng Fu reports financial support was provided by Xinjiang Uygur Autonomous Region Department of Science and Technology. Xuefeng Fu reports financial support was provided by National Natural Science Foundation of China. If there are other authors, they declare that they have no known competing financial interests or personal relationships that could have appeared to influence the work reported in this paper.

## Data Availability

The original contributions presented in the study are included in the article, further inquiries can be directed to the corresponding author. The mRNA expression profiles of 18 Xinjiang goats skin tissues in this paper have been deposited in the China National Center for Bioinformation (ID: OMIX014908).

## Appendix Supplementary materials

Image, image 1 dummy alt text

Image, image 2 dummy alt text

Image, image 3 dummy alt text

Image, image 4 dummy alt text

Image, image 5 dummy alt text

Supplementary Materials The following supporting information can be downloaded at: Table S1. Interference fragments and cloning primers of the *ELOVL3* gene; Table S2. Sequence information of qPCR primer; Table S3. RNA-Seq quality control result; Figure S1. WGCNA Analysis; Figure S2. Co-expression interaction networks of the top 10 hub genes in each significant WGCNA module; Figure S3. Expression patterns of the top 10 hub genes from five significant WGCNA co-expression modules across six months; Figure S4. Functional Annotation Analysis of Candidate Genes; Figure S5. Construction of *ELOVL3* gene overexpression vector. Image, application 6
