# Supplementary material for: Dynamic analysis of skin transcriptome in Xinjiang goats and functional verification of ELOVL3 in dermal papilla cell proliferation
Source: Vet Anim Sci. 2026 Aug 24;33:100834. doi: 10.1016/j.vas.2026.100834 (PMC13524765; doi:10.1016/j.vas.2026.100834)
Supplement: Supplementary file 1 [file mmc6.docx]

**Table S1**. Interferences fragment and cloning primers of the *ELOVL3* gene

| **Sequence Name** | **Sequence (5’-3’)** |
| --- | --- |
| ELOVL3-si-381 | F: GCAGAGACCUCUCAUCCUUTT |
|  | R: AAGGAUGAGAGGUCUCUGCTT |
| ELOVL3-si-586 | F: GCCUUCAUCAUCCUGCGUATT |
|  | R: UACGCAGGAUGAUGAAGGCTT |
| ELOVL3-si-648 | F: GCUGGUGUACUCGAGCUUUTT |
|  | R: AAAGCUCGAGUACACCAGCTT |
| si-NC | F: UUCUCCGAACGUGUCACGUTT |
|  | R: ACGUGACACGUUCGGAGAATT |
| *ELOVL3* | F:GCGTTTAAACTTAAGCTTGGTACCGAGCTCGGATCCATGGTCACAACAGTGAATACCTCAGATGA |
|  | R:GAGCGGCCGCCACTGTGCTGGATATCTGCAGAATTCTCACTGGCTCTTGGTCTTGGCTTTGATCT |

**Table S2.** Sequence information of qPCR primer

| **Gene Name** | **Sequence （5’- 3’）** | **Product Length/bp** | | **Gene ID** |
| --- | --- | --- | --- | --- |
| *ELOVL3* | F:CACTGTCGGTATCCTGGCTT | 182 | XM_005698356 | |
|  | R:CTCCAACCACTCACTGGCTC |  |  |  |
| *GAPDH* | F:GGCGTGAACCACGAGAAGTAT R:ATGGCGTGGACAGTGGTCAT | 143 | XM_005680968.3 | |

**Table S3.** RNA-Seq quality control result

| **Sample** | **Obtained Reads** | **OBtained Base(bp)** | **Q20(%)** | **Q30(%)** | **GC(%)** |
| --- | --- | --- | --- | --- | --- |
| X1.1 | 34,778,481 | 10,401,112,616 | 98.24 | 95.37 | 51.53 |
| X1.2 | 34,349,387 | 10,271,118,106 | 98.23 | 95.38 | 51.25 |
| X1.3 | 34,700,852 | 10,375,078,432 | 98.00 | 95.02 | 52.05 |
| X3.1 | 34,462,078 | 10,307,012,004 | 98.29 | 95.48 | 51.34 |
| X3.2 | 34,839,078 | 10,421,435,302 | 98.2 | 95.28 | 52.14 |
| X3.3 | 35,255,738 | 10,542,497,832 | 98.26 | 95.45 | 51.30 |
| X5.1 | 34,500,006 | 10,318,573,870 | 98.26 | 95.44 | 51.26 |
| X5.2 | 35,498,411 | 10,615,649,878 | 98.25 | 95.42 | 51.50 |
| X5.3 | 34,883,574 | 10,432,379,512 | 98.27 | 95.43 | 51.36 |
| X7.1 | 34,172,079 | 10,222,871,178 | 98.33 | 95.45 | 51.52 |
| X7.2 | 34,325,143 | 10,268,351,164 | 98.28 | 95.36 | 51.05 |
| X7.3 | 31,912,738 | 9,541,448,714 | 98.34 | 95.67 | 51.98 |
| X9.1 | 35,045,744 | 10,480,769,262 | 98.24 | 95.37 | 51.60 |
| X9.2 | 34,786,693 | 10,403,819,352 | 98.24 | 95.34 | 51.39 |
| X9.3 | 34,391,636 | 10,283,949,836 | 98.21 | 95.35 | 51.24 |
| X11.1 | 35,102,642 | 10,498,895,946 | 98.30 | 95.55 | 51.65 |
| X11.2 | 34,230,353 | 10,238,258,504 | 98.27 | 95.39 | 51.01 |
| X11.3 | 34,604,013 | 10,349,128,944 | 98.32 | 95.59 | 51.30 |

Note: Obtained Reads: The total number of sequencing reads obtained from the sample. Obtained Bases: The total number of base pairs obtained from the sequencing reads. Q20 (%): The percentage of base calls with a quality score ≥ 20. Q30 (%): The percentage of base calls with a quality score ≥ 30. GC (%): The percentage of guanine (G) and cytosine (C) nucleotides in the sample.

**Table S4.** Transcriptome mapping result statistics

| **Sample** | **Total Reads** | **Paired in squencing** | **Properly paired** | **Reads Map to '+'** | **Reads Map to '-'** | **MapQ** |
| --- | --- | --- | --- | --- | --- | --- |
| X1.1 | 73109935 | 67177985 | 64202904  (95.57%) | 33725625  (46.13%) | 33452360  (45.76%) | 1083630  (1.61%) |
| X1.2 | 71942859 | 66339526 | 63761974  (96.11%) | 33309447  (46.29%) | 33030079  (45.91%) | 778480  (1.17%) |
| X1.3 | 72614978 | 66625676 | 63656668  (95.54%) | 33544559  (46.20%) | 33081117  (45.56%) | 786568  (1.18%) |
| X3.1 | 71896433 | 66447333 | 63351756  (95.34%) | 33344891  (46.38%) | 33102442  (46.04%) | 1148804  (1.73%) |
| X3.2 | 72528934 | 67310836 | 64555774  (95.91%) | 33796030  (46.60%) | 33514806  (46.21%) | 943136  (1.40%) |
| X3.3 | 73611837 | 68050764 | 65611450  (96.42%) | 34122847  (46.36%) | 33927917  (46.09%) | 627282  (0.92%) |
| X5.1 | 70821680 | 66474596 | 63509674  (95.54%) | 33366979  (47.11%) | 33107617  (46.75%) | 1084668  (1.63%) |
| X5.2 | 73705742 | 68444637 | 65509130  (95.71%) | 34350677  (46.61%) | 34093960  (46.26%) | 994982  (1.45%) |
| X5.3 | 72256447 | 66870430 | 63390986  (94.80%) | 33567305  (46.46%) | 33303125  (46.09%) | 1401180  (2.10%) |
| X7.1 | 71957364 | 66209123 | 63242756  (95.52%) | 33243645  (46.20%) | 32965478  (45.81%) | 1218668  (1.84%) |
| X7.2 | 72762896 | 66484759 | 64002348  (96.27%) | 33396233  (45.90%) | 33088526  (45.47%) | 727826  (1.09%) |
| X7.3 | 67184395 | 61490389 | 58574732  (95.26%) | 30856267  (45.93%) | 30634122  (45.60%) | 1072640  (1.74%) |
| X9.1 | 74855059 | 67736356 | 64932882  (95.86%) | 34010061  (45.43%) | 33726295  (45.06%) | 910780  (1.34%) |
| X9.2 | 72937425 | 67114863 | 63834072  (95.11%) | 33689725  (46.19%) | 33425138  (45.83%) | 1322380  (1.97%) |
| X9.3 | 71543996 | 65927864 | 63210870  (95.88%) | 33100437  (46.27%) | 32827427  (45.88%) | 839966  (1.27%) |
| X11.1 | 74101989 | 67954356 | 65500190  (96.39%) | 34100693  (46.02%) | 33853663  (45.69%) | 673946  (1.00%) |
| X11.2 | 71016848 | 66137256 | 63358294  (95.80%) | 33187623  (46.73%) | 32949633  (46.40%) | 1002204  (1.52%) |
| X11.3 | 72645385 | 66867181 | 64482242 (96.43%) | 33544450  (46.17%) | 33322731  (45.87%) | 609966 (0.91%) |

Note: Paired in sequencing: Number of paired-end read pairs obtained from sequencing. Properly paired: Number of read pairs correctly aligned to the reference genome, meeting paired-end orientation and insert size requirements, with their percentage relative to total paired-end reads. Reads Map to '+': Number of reads aligned to the forward (+) strand of the reference genome, with their percentage relative to total clean reads. Reads Map to '-': Number of reads aligned to the reverse (-) strand of the reference genome, with their percentage relative to total clean reads. MapQ: Number of abnormally aligned reads, with their percentage relative to total clean reads.

**Figure S1**. WGCNA Analysis

(A) Determination of the soft threshold. (B) Construct a hierarchical clustering tree using the TOM values between genes.

**Figure S2.** Co-expression interaction networks of the top 10 hub genes in each significant WGCNA module

(A) MEblue. (B) MEcyan. (C) MEdarkgreen. (D) MEgreen. (E) MEturquoise. The node color gradient from red to yellow indicates decreasing hub scores, and numbers on dotted lines represent pairwise gene connectivity weights.

**Figure S3.** Expression patterns of the top 10 hub genes from five significant WGCNA co-expression modules across six months

(A) MEblue. (B) MEcyan. (C) MEdarkgreen. (D) MEgreen. (E) MEturquoise. The y-axis represents gene expression levels normalized as log₂ (cpm), and the x-axis indicates sampling months throughout the year.

**Figure S4**. Functional Annotation Analysis of Candidate Genes

(A) Bubble chart for GO enrichment analysis. The horizontal axis represents the enrichment intensity, and the vertical axis represents the GO term. (B) Bubble chart for KEGG enrichment analysis. The horizontal axis represents the enrichment intensity, and the vertical axis represents the pathway.

**Figure S5.** Construction of *ELOVL3* gene overexpression vector

(A) Results of double enzyme digestion. (B) pCDNA3.1(+)-ELOVL3.
